# Effect of Electrode Material on the Crystallization of GeTe Grown by Atomic Layer Deposition for Phase Change Random Access Memory

**DOI:** 10.3390/mi10050281

**Published:** 2019-04-27

**Authors:** Seung Ik Oh, In Hyuk Im, Chanyoung Yoo, Sung Yeon Ryu, Yong Kim, Seok Choi, Taeyong Eom, Cheol Seong Hwang, Byung Joon Choi

**Affiliations:** 1 Department of Materials Science and Engineering, Seoul National University of Science and Technology (Seoultech), Seoul 01811, Korea; kg5176@naver.com (S.I.O.); dhfnvl1@naver.com (I.H.I.); tjddus0205@naver.com (S.Y.R.); kimyonggg@naver.com (Y.K.); gkop0820@naver.com (S.C.); 2 Department of Materials Science and Engineering, and Inter-University Semiconductor Research Center, Seoul National University, Seoul 08826, Korea; cyyoo0117@snu.ac.kr (C.Y.); cheolsh@snu.ac.kr (C.S.H.); 3 Division of Advanced Materials, Korea Research Institute of Chemical Technology (KRICT), 141 Gajeong-Ro, Yuseong-Gu, Daejeon 34114, Korea; eomt@krict.re.kr

**Keywords:** phase change random access memory, crystallization behavior, Johnson–Mehl–Avrami kinetics, electrode interfacial layer effect

## Abstract

The electrical switching behavior of the GeTe phase-changing material grown by atomic layer deposition is characterized for the phase change random access memory (PCRAM) application. Planar-type PCRAM devices are fabricated with a TiN or W bottom electrode (BE). The crystallization behavior is characterized by applying an electrical pulse train and analyzed by applying the Johnson–Mehl–Avrami kinetics model. The device with TiN BE shows a high Avrami coefficient (>4), meaning that continuous and multiple nucleations occur during crystallization (set switching). Meanwhile, the device with W BE shows a smaller Avrami coefficient (~3), representing retarded nucleation during the crystallization. In addition, larger voltage and power are necessary for crystallization in case of the device with W BE. It is believed that the thermal conductivity of the BE material affects the temperature distribution in the device, resulting in different crystallization kinetics and set switching behavior.

## 1. Introduction

Given the recent ever-increasing growth of the memory market, research on low-power, high-speed, and high-density nonvolatile memory for the next generation is being actively conducted. Phase change random access memory (PCRAM)—one of the next-generation memory candidates—utilizes a reversible phase change between amorphous and crystalline phases through Joule heating generated by the current passing through a nanoscale device [1,2,3]. PCRAM has been seriously considered as a new storage-class memory and can be used for 3D Xpoint memory owing to its high read/write speed, high density, and nonvolatile characteristics [4,5]. In addition, PCRAM can provide a feasible artificial synapse for neuromorphic hardware systems due to its multilevel memory functionality and high reliability [6,7,8,9]. Despite the significant improvement made in the device fabrication technique and material innovation during the past decades, a too high operation current has been the major hurdle for high-density low-power devices. Between the two memory operations, i.e., switching from the amorphous to crystalline state (set) and that from the crystalline to amorphous state (reset), reset takes higher current consumption because it involves the (local) melt-quenching process of the phase change material. Therefore, improving the thermal efficiency by increasing thermal insulation and more localized heating has been pursued, and has been accomplished through the device structure innovation and modification of the electrode material [10,11,12]. Besides, the crystallization process (set switching) is crucial because the crystallization of the amorphous region is the slowest process, and hence, will determine the maximum speed in PCRAM as nonvolatile memory or neuromorphic system applications [6,13]. Multilevel switching can also be achieved by controlling the crystallization process by using the engineering programming pulse scheme or varying the material stack [14,15,16]. Since the crystallization process is also thermally driven, the heat generation and dissipation when the set current flows are also critical for confirming the device performance, which is critically related to the contacting electrodes. Between the top and bottom electrodes, the bottom electrode (BE) has higher relevance in this regard because the local phase change occurs at the interface with BE. The different BE materials can have different interface energies, which influences the heterogeneous nucleation of the crystalline seed. They can also have different thermal conductivities, which can influence the thermal efficiency and local temperature, which critically increases the overall nucleation and growth of the crystalline phase. Therefore, it is necessary to study the effect of electrode material on the device characteristics to understand the crystallization process in PCRAM operation.

In this study, an atomic-layer-deposited GeTe-based PCRAM device was fabricated on TiN and W as BE, and the effect of electrode material on the crystallization of the GeTe phase-changing material was investigated. GeTe or Ge–Sb–Te phase-changing material, fabricated via atomic layer deposition (ALD), shows excellent step coverage even in a very-high-aspect-ratio (20:1) trench structure [17,18,19,20]. In addition, the conformal deposition will guarantee uniformity in electrical properties, including the crystallization process, because the void and adhesion issue can be avoided. While the contact-hole-type PCRAM has higher thermal efficiency, this study adopted a simple planar-type cell (or mushroom cell) with two different BE materials plugged into a bottom contact hole to simplify the integration process. A common TiN top electrode (TE) was adopted for both devices. 

By measuring the current–voltage (I–V), resistance–voltage (R–V), and resistance–time (R–t) curves of the devices, Johnson–Mehl–Avrami (JMA) kinetics were applied to analyze the nucleation and growth behavior of a GeTe crystal. Three-dimensional hemispherical crystalline growth with continuous nucleation, and possibly three-dimensional crystalline growth with retarded nucleation models, is suggested for devices with TiN and W BE being used. An electro-thermal simulation was conducted to understand the thermal effect of BE material in determining the crystallization process. 

## 2. Materials and Methods 

**Figure 1:**
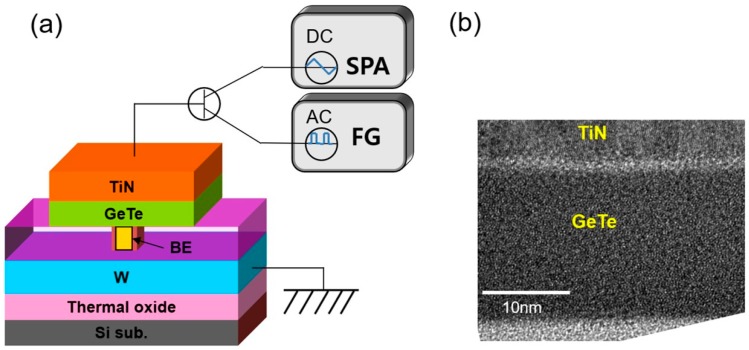
(**a**) Schematics of the fabricated planar-type phase change memory device (TiN/GeTe/TiN (TGT) and TiN/GeTe/W (TGW)) and electrical measurement system (SPA: semiconductor parameter analyzer; FG: arbitrary function generator). (**b**) Cross-sectional TEM image of TiN/GeTe stack in TGT device.

Planar-type PCRAM devices were fabricated as shown in Figure 1a. Two types of BE—TiN and W—were formed as a contact hole surrounded by a SiO_2_ layer, whose diameter was varied from 50 nm to 2 μm. The devices with BE diameter of 220 nm were used to examine the detailed switching performances of the two types of BEs. A 20-nm-thick GeTe layer as a phase-changing material was grown by thermal ALD. GeTe films were deposited using a shower-head-type ALD reactor (CN-1, Atomic-premium). The substrate temperature was maintained at 70 °C. The precursors of Ge and Te were Ge(N(Si(CH_3_)_3_)_2_)_2_ and ((CH_3_)_3_Si)_2_Te, respectively. The details of the ALD process for GeTe can be found in previous literature [18,19]. Sputter-grown 100-nm-thick TiN was formed as a TE. Devices with TiN/GeTe/TiN (TGT) and TiN/GeTe/W (TGW) stacks were finally prepared for the analysis. 

As shown in Figure 1a, a DC I–V measurement was performed using a semiconductor parameter analyzer (SPA, Agilent, HP-4155A model, Santa Clara, CA, USA). The electric pulse measurement for the R–V curve was performed using an arbitrary function generator (FG, Tektronix, AFG-3012 model, Beaverton, OR, USA). The electrical tests were performed at room temperature (~25 °C) for both devices. The common W blanket layer (150 nm-thick) was placed below the 100-nm-deep BE holes and grounded in all measurements. Transmission electron microscopy (TEM, JEM-2100F, JEOL, Tokyo, Japan), combined with energy-dispersive X-ray spectroscopy (EDS), was used to observe the cross-section of the device. Figure 1b shows a high-resolution TEM image, focusing on the GeTe layer of the TGT device. The as-grown GeTe film was composed of a fully amorphous phase and uniformly deposited by ALD. The composition of the GeTe film was close to that of Ge_0.6_Te_0.4_, which was confirmed by X-ray fluorescence spectroscopy and TEM-EDS. It was revealed that the as-grown GeTe films were amorphous with a tetrahedral Ge coordination of a uniform mixture of (major) Ge–Te and (minor) Ge–Ge bonds, according to the X-ray absorption fine structure analysis [20].

## 3. Results

**Figure 2:**
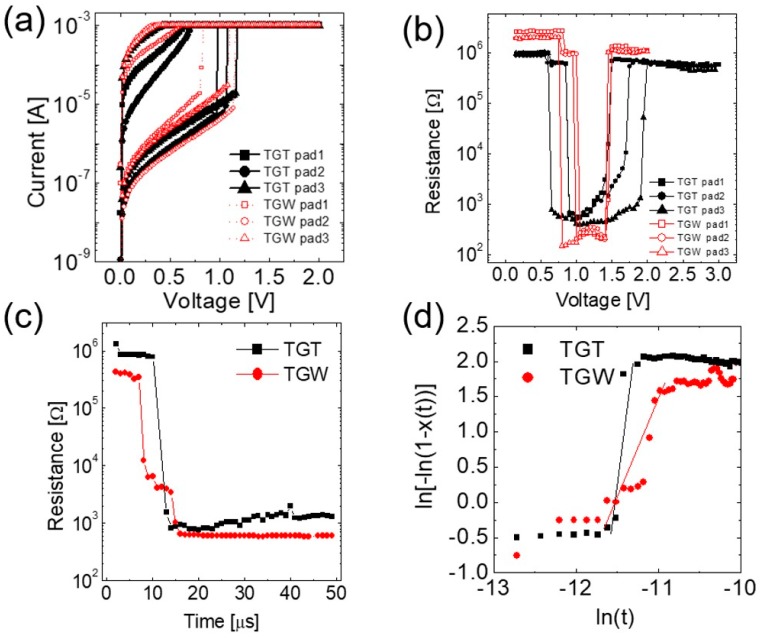
(**a**) Set switching of TGT and TGW devices induced by DC I–V (pad number means the different memory cells in the device). (**b**) Set and reset switching of TGT and TGW devices representing R–V curves induced by AC pulse with 1 μs width. (**c**) Voltage pulse-induced set transition of TGT and TGW devices, where consecutive voltage pulse train with 1 μs width (0.45 V for TGW and 0.6 V for TGT) was used. (**d**) Set transitions of both devices were expressed as a JMA plot.

Figure 2a shows the set switching characteristics tested by the DC I–V sweep of the TGT and TGW devices, respectively, from the pristine state. As can be understood from Figure 1b, the pristine devices had an amorphous structure of the GeTe layer, and initially showed the reset state. The set switching occurred at 1.1 ± 0.1 V (1.0 ± 0.2 V) for the TGT (TGW) device. The V_set_ (set voltage) required for GeTe crystallization by DC bias was similar in both devices. The pulse-type set and reset switching were also tested by providing writing and reading voltage pulse trains (pulse width = 1 μs), as shown in the R–V characteristics in Figure 2b. Here, V corresponds to the writing pulse voltage, while the reading voltage was fixed at 0.3 V. The TGW device shows a sharp transition for the first set and subsequent reset transitions, while the TGT device showed a similar sharp set transition and a gradual reset transition. According to the AC measurement, V_set_ and V_reset_ (reset voltage) for TGT (TGW) were 0.8 ± 0.1 V (0.9 ± 0.1 V) and 1.7 ± 0.2 V (1.47 ± 0.03 V), respectively. The much higher thermal conductivity of W BE in TGW, compared to TiN BE in TGT, may induce a lower thermal efficiency during the reset transition, which could be the reason for the gradual reset behavior. However, this topic will not be discussed in detail as this study focuses on the set switching behavior depending on the BE types. Therefore, in what follows, the pulse-type-set switching behaviors are scrutinized. 

To understand the crystallization kinetics in the devices, the R–t characteristics for set switching were examined. Figure 2c exhibits the typical set switching operation by applying an electrical pulse with 1 μs width and smaller voltage height than V_set_ (0.45 V for TGW and 0.6 V for TGT) to observe the variation in resistance over time during crystallization. The x-axis of Figure 2c represents the cumulative pulse time. The chosen V_set_ was the minimum voltage for the set switching for the given pulse width. The change in resistance of the TGT device occurred abruptly, while that in the TGW device occurred via the intermediate states. 

The JMA kinetics are applied to examine the behavior of the crystalline growth of each device shown in Figure 2c [9]. For this analysis, the crystallization fraction, *x*(*t*), of the phase-changing material, is assumed to be related to the resistance of the memory cell at a certain time *t* (*R*(*t*)) according to Equation (1).

(1) $$x(t)=\frac{R_{amor.}-R(t)}{R_{amor.}-R_{cry.}}$$

where *R_amor_*_._ and *R_cry_*_._ are the values of resistance in the fully amorphous and crystalline phases. The JMA kinetics is an equation for kinetics in the phase transition mechanism, generally expressed as shown in Equation (2) [21,22].

(2) $$x(t)=1-\exp[-{\left(kt\right)}^{n}]$$

where *n* is the Avrami coefficient and *k* is the effective rate constant. Equation (2) can be modified to deal with the R–t curve, as follows: [21,22,23]

(3) $$\ln[\ln[\frac{1}{1-x(t)}]]=n\ln t+n\ln k$$

According to Equation (3), *n* can be acquired from the slope of ln(t) versus ln[−ln(1−*x*(t))] plot. The Avrami coefficient, *n*, can be expressed as the sum of two elements, *n* = *a* + *b*, where *a* indicates the nucleation index, *b* indicates the dimensionality of the growth (such as *b* = 1, 2, or 3 for one-, two-, or three-dimensional growth, respectively, where nuclei grow like needles, disks, or spheres) [21,22,23,24,25].

The variation in the resistance with cumulative time of voltage pulse (Figure 2c) can be represented as a JMA plot (Figure 2d). After the incubation time (~9–10 μs), an abrupt increment in *x*(t) is observed in the double log scale in the TGT device. As mentioned above, the Avrami coefficient (*n*) can be determined from the slope of linear fitting. In case of the TGT device, an n value of ~5.4 in the range of 9–13 μs is acquired after the incubation time. Generally, the crystalline nuclei have three-dimensional shapes within the dome-shaped amorphous region in the planar-type device during the set (recrystallization) process, suggesting that *b* ≈ 3. Therefore, the experimental results for *n* = 5.4 suggest that *a* ≈ 2.4. A previous analysis conducted using the JMA model revealed that *n* = 4 for a constant nucleation rate and *n* > 4 for an increasing nucleation rate [26,27,28]. Therefore, the recrystallization in the TGT device can be identified as follows: once the nucleation is initiated after the incubation time, the number of nuclei increase with time and grow three-dimensionally to form a hemispherical active region. 

In contrast, in case of a TGW device, an *n* value of ~3 is acquired in the linear range of 5–15 μs in Figure 2d. *n* = 3 is interpreted as an indicative of the retarded nucleation, i.e., once one nucleus forms, it keeps growing while the additional nucleation is suppressed [27,28]. This is consistent with the subsequent analysis based on the electro-thermal simulations.

**Figure 3:**
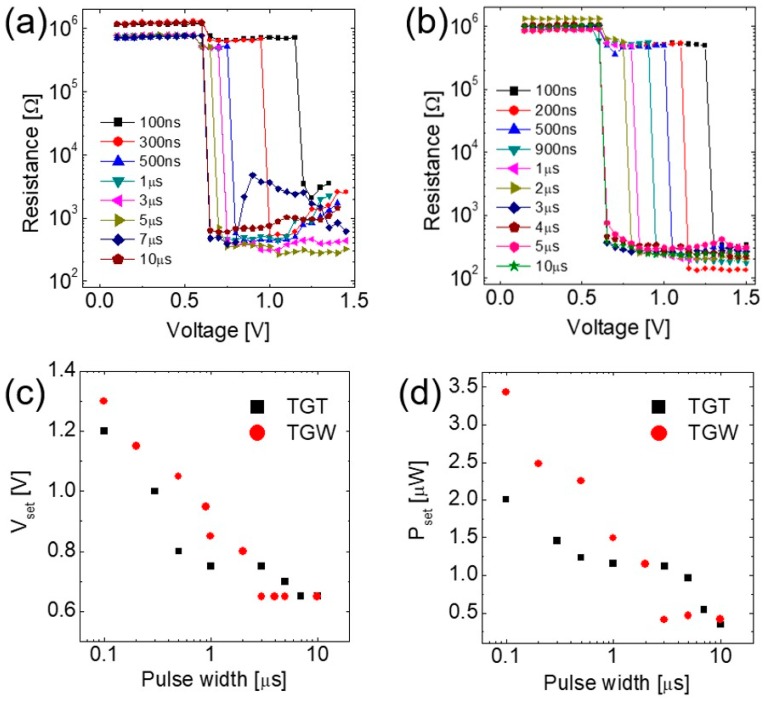
R–V curves obtained by changing the pulse width: (**a**) TGT and (**b**) TGW devices. Variation in (**c**) V_set_ and (**d**) P_set_ as a function of pulse width for TGT and TGW devices.

Next, variation in the resistance of the devices as a function of pulse width was examined by applying incremental voltage pulses with pulse width ranging from 100 ns to 10 μs, as shown in Figure 3a,b. These R–V curves show that the abrupt set transition occurs at V_set_, which rapidly decreases with an increasing pulse width in both TGT and TGW devices, as shown in Figure 3c. The V_set_ of the TGW device is slightly higher than that of the TGT device when the pulse width is short (<1 μs), but rapidly decreases and saturates by applying a longer pulse (>3 μs). This stronger time-dependency of the TGW device is more evident when the power for the set switching (P_set_ = V_set_^2^/R) is calculated and compared, as shown in Figure 3d. Fifty to seventy percent higher power is required for the set switching of the TGW device by using 100–500 ns of pulse width, despite a marginal difference in V_set_ (0.1–0.3 V). Such a strong time-dependency and high power for the set switching (i.e., crystallization) of the TGW device are attributed to heat dissipation via the W BE. In other words, a higher power (for Joule heating) is necessary to invoke the nucleation and growth for the TGW device under a shorter pulse width, because a rapid rate of temperature increase by a shorter pulse induces larger heat dissipation. On the other hand, the TGT device may not suffer from severe heat dissipation, and thus, the requirement of power for the set switching shows weaker time-dependency. 

**Figure 4:**
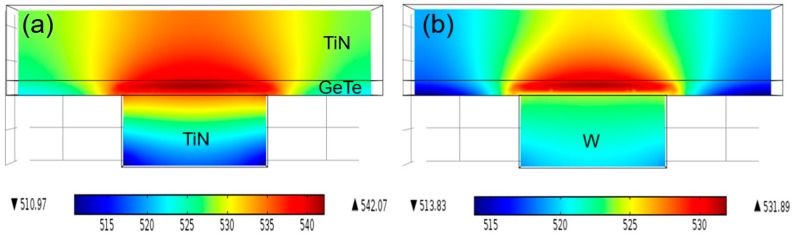
Temperature distribution (in Kelvin) in the device by electro-thermal simulation: (**a**) TGT and (**b**) TGW devices.

An electro-thermal simulation was performed using the Joule heating model (COMSOL, Multiphysics, 4.3b) to calculate the temperature distribution in the devices. A planar-type device structure was constructed with the TGT and TGW stacks. The large difference in the thermal conductivities of TiN (29 W/(m·K)) and W (170 W/(m·K)) was noted in the simulation. In this simulation, a sufficiently large set current of 500 μA was assumed to guarantee the high enough temperature required for recrystallizing GeTe in a short period. The crystallization temperature of bulk GeTe is ~448 K and can be increased up to ~510 K at the nanoscale [29]. The material parameters were selected from the material library in the program. The bottom and pad interfaces were held at an isothermal temperature of 300 K. Figure 4a,b exhibit the simulation results of the temperature distribution of the TGT and TGW devices, respectively. The difference in the temperature profile can be observed in the contact region between GeTe and BE. The TGW device has a lower maximum temperature and more concentrated temperature distribution compared to the TGT device. It is considered that heat dissipation occurs toward the BE due to the larger thermal conductivity of W, which results in less residual heat remaining in the active region of the TGW device. In contrast, the lower heat loss through the BE due to the lower thermal conductivity of TiN BE results in a higher peak temperature as well as a wider distribution of the high-temperature zone within the GeTe layer. A wider distribution of the high-temperature zone in the TGT device enhances the probability of multiple nucleations, which is consistent with the higher n value of the JMA model, as shown in Figure 2d. In contrast, the rapid heat dissipation effect near GeTe/W in the TGW device may hinder the additional nucleation, and only one or two nuclei at the narrow highest temperature zone became dominant during the set switching.

## 4. Discussion

**Figure 5:**
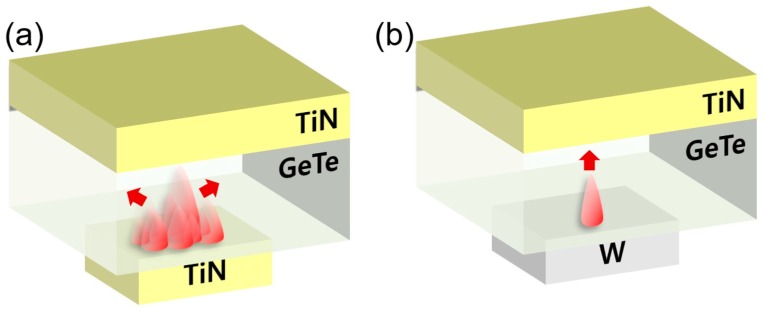
Schematics of the crystallization model suggested by considering JMA kinetics and simulation: (**a**) TGT (crystalline growth with multiple nuclei) and (**b**) TGW (crystalline growth with one nucleus).

Figure 5a,b show the schematic diagrams of the crystallization model for the TGT and TGW devices suggested by the JMA kinetics and simulation. The large difference in the thermal conductivity of TiN and W may invoke disparate crystallization kinetics and set switching. For example, the resistance state is unstable and even increased after set switching, as shown in Figure 2b and Figure 3a, in the TGT device. This can be understood from the excessive heating caused by the thermal boundary resistance of GeTe/TiN, which may induce an unwanted local melt-quench effect at especially smaller crystallites. Lee et al. reported that an additional thermal boundary resistance might become important due to the electron–phonon coupling at GST/TiN interfaces under higher temperature than the Debye temperature of TiN (580 K) [30]. It is considered that the lower thermal conductivity and electron–phonon coupling can increase the overall resistance of the GeTe/TiN interface slightly above the crystallization temperature. In contrast, the fluent heat dissipation at the GeTe/W interface does not induce such an adverse effect after the set switching, and thus, a stable set state can be achieved. Those crystallization models should be further investigated by other methods, such as TEM or local conductivity mapping.

## 5. Conclusions

The effects of TiN and W BE on the recrystallization behavior of the GeTe phase-changing material grown by ALD were examined by DC I–V and pulse-switching experiments to test a PCRAM cell. The DC I–V and voltage pulse were introduced into the devices for the set transition and their responses were measured by their resistance states. The set transition was analyzed according to the JMA crystallization kinetics. The TGT device had a longer incubation time, and showed multiple nucleations and three-dimensional crystalline growth. In contrast, the TGW device had a shorter incubation time and only one (or smaller number) nuclei nucleated and kept growing. This difference could be mainly attributed to the different temperature distributions of the two types of devices during the set switching. The much lower thermal conductivity of TiN compared to that of W resulted in a higher temperature and wider distribution of the hot zones, which may induce multiple nuclei formation. In contrast, the higher thermal conductivity of W BE induced more localized hot zone distribution, which could contribute to the more localized recrystallization. The more localized and single recrystallized material in the TGW device induced higher stability of the set state compared to the TGT device.
